# Hematopoietic Protein-1 Regulates the Actin Membrane Skeleton and Membrane Stability in Murine Erythrocytes

**DOI:** 10.1371/journal.pone.0054902

**Published:** 2013-02-12

**Authors:** Maia M. Chan, Jason M. Wooden, Mark Tsang, Diana M. Gilligan, Dinesh K. Hirenallur-S, Greg L. Finney, Eric Rynes, Michael MacCoss, Julita A. Ramirez, Heon Park, Brian M. Iritani

**Affiliations:** 1 Department of Comparative Medicine, University of Washington, Seattle, Washington, United States of America; 2 Puget Sound Blood Center, Seattle, Washington, United States of America; 3 Department of Genome Sciences, University of Washington, Seattle, Washington, United States of America; Université de Genève, Switzerland

## Abstract

Hematopoietic protein-1 (Hem-1) is a hematopoietic cell specific member of the WAVE (Wiskott-Aldrich syndrome verprolin-homologous protein) complex, which regulates filamentous actin (F-actin) polymerization in many cell types including immune cells. However, the roles of Hem-1 and the WAVE complex in erythrocyte biology are not known. In this study, we utilized mice lacking Hem-1 expression due to a non-coding point mutation in the *Hem1* gene to show that absence of Hem-1 results in microcytic, hypochromic anemia characterized by abnormally shaped erythrocytes with aberrant F-actin foci and decreased lifespan. We find that Hem-1 and members of the associated WAVE complex are normally expressed in wildtype erythrocyte progenitors and mature erythrocytes. Using mass spectrometry and global proteomics, Coomassie staining, and immunoblotting, we find that the absence of Hem-1 results in decreased representation of essential erythrocyte membrane skeletal proteins including α- and β- spectrin, dematin, p55, adducin, ankyrin, tropomodulin 1, band 3, and band 4.1. *Hem1^−/−^* erythrocytes exhibit increased protein kinase C-dependent phosphorylation of adducin at Ser724, which targets adducin family members for dissociation from spectrin and actin, and subsequent proteolysis. Increased adducin Ser724 phosphorylation in *Hem1^−/−^* erythrocytes correlates with decreased protein expression of the regulatory subunit of protein phosphatase 2A (PP2A), which is required for PP2A-dependent dephosphorylation of PKC targets. These results reveal a novel, critical role for Hem-1 in the homeostasis of structural proteins required for formation and stability of the actin membrane skeleton in erythrocytes.

## Introduction

Erythrocytes are unique cells in that they have a biconcave shape, can repeatedly deform and regain their shape, and can withstand turbulence and shear forces exerted upon them by fine capillary vessel walls [1]. These complimentary features of strength and flexibility are intrinsic to the interplay between their fluid cell membrane and the resilient yet pliable lattice structure of the membrane skeleton [1], [2]. The importance of maintaining a strong yet flexible erythrocyte membrane skeleton is underscored by the fact that the majority of erythrocyte disorders, which are collectively the most common inherited disorders in humans worldwide [1], are caused by mutations in genes encoding essential membrane structural proteins.

Numerous studies have resulted in the identification of a set of core components of the erythrocyte membrane skeleton that allow for their unique mechanical characteristics. The plasma membrane consists of a phospholipid and cholesterol bilayer interspersed with critical transmembrane proteins such as band 3 and glycophorins A and C (see [3] for review). These transmembrane proteins form two major intracellular complexes called the “Ankyrin Complex” (which links membrane bound glycophorin A, band 3, Rh, and CD47 to intracellular ankyrin and protein 4.2) and the “Junctional Complex” (which links transmembrane glycoprotein C, band 3, Rh, and Glut1 to intracellular dematin, P55, protein 4.1, tropomodulin, tropomyosin, and actin). The ankyrin and junctional complexes further form “vertical” attachments or bridges to the underlying membrane skeleton, which consists of a precise hexagonal lattice of complexes containing short filaments of 12–18 actin monomers connected by longer flexible helices of α- and β- spectrin tetramers [2], [3], [4], [5]. The associations between spectrin and actin with the junctional and ankyrin complexes are critical for allowing erythrocytes to maintain their shape and to withstand physical forces associated with transport in circulation. Disruption of these intricate interactions can result in erythrocyte fragmentation, removal by the spleen, and hemolytic anemia [1].

Because of its central location and multiple interactions with membrane skeletal proteins, the appropriate polymerization and organization of actin is paramount to maintaining the strength and deformability of erythrocytes. The formation of actin filaments (F-actin) is regulated by the actin regulatory complex (ARP2/3), which stimulates monomeric globular actin (G-actin) to polymerize at both the fast growing barbed end and slower growing pointed end [6]. The newly formed actin filaments are further stabilized into fixed lengths by a number of actin capping proteins including adducin, tropomyosin, tropomodulin, dematin, capZ, p55, and protein 4.1R [4], [7], which act to prevent actin monomers from associating or disassociating. The importance of stabilizing actin filaments in erythrocytes is underscored by the observations that mutations or deletions in genes encoding adducin [8], dematin [2], tropomyosin [9], tropomodulin [10], or protein 4.1 [11] all lead to red blood cell diseases associated with increased erythrocyte fragility and anemia (see [1] for review).

In some cell types such as T cells, the signaling pathways that stimulate reorganization of the actin cytoskeleton are partially understood [12]. However, in erythrocytes there is very little known about the intracellular signaling pathways that control the formation and stability of the erythrocyte membrane skeleton. Recent studies suggest that some members of the Rho family of GTPases (which include Rac -1 and -2, Cdc42, and Rho -A, -H, and -G in hematopoietic cells) may be involved in actin polymerization and reorganization in erythrocytes [13]. In human erythrocytes, RhoA has been found to translocate from the cytosol to the membrane, potentially resulting in active signaling modules [14]. In mice, conditional disruption of Rac1 and Rac2 results in microcytic anemia characterized by gaps in the actin membrane skeleton, irregularity of the spectrin scaffold, decreased deformability, and decreased representation of essential skeletal proteins including α- and β- spectrin and adducin [3]. Rac GTPases are activated in response to extracellular signals, which stimulate guanine nucleotide exchange factors to catalyze the exchange of bound GDP for GTP on Rac. GTP-bound activated Rac then interacts with PAK (P21-activated kinase), DIAP3 (Diaphanous homolog 3), and an assembly of proteins collectively known as the WAVE complex, which stimulate ARP2/3 to initiate F-actin polymerization [15], [16]. The WAVE complex is a pentameric heterocomplex consisting of WAVE (WAVE -1, -2, or -3); Abi (Abelson-interacting protein, Abi -1 or -2); Hem [hematopoietic protein Hem-2 (also known as Nap1 (Nck-associated protein 1, or NckAP1)) or Hem-1 (also known as NckAP-1like)]; Sra1 (also known as CYFIP1); and HSPC300 (hematopoietic stem/progenitor cell protein 300) [16], [17], [18], [19].

Whereas the majority of WAVE complex subunits are ubiquitously expressed, Hem-1 is found predominantly in hematopoietic cells [16], [20], suggesting a specialized role for Hem-1 in regulating the biology of hematopoietic cells. We recently generated an N-ethyl-N-nitrosourea (ENU) mutant mouse that contains a single, non-coding point mutation in the *Hem1* gene, which results in the absence of Hem-1 protein [16]. Hem-1 null mice were noted to have a number of defects in immune cell development and function due in part to destabilization and degradation of WAVE complex proteins, which resulted in impaired F-actin polymerization and formation of the actin cytoskeleton [16]. Here we utilized Hem-1 null mice to show that loss of Hem-1 also results in microcytic, hypochromic anemia characterized by abnormal F-actin condensation, altered representation and phosphorylation of essential junctional complex proteins, altered erythrocyte morphology, and significantly reduced erythrocyte lifespan. These results identify a novel role for Hem-1, and perhaps the WAVE complex, in controlling the formation and stability of the actin membrane skeleton in red blood cells.

## Materials and Methods

### Mice

*Hem1^−/−^* mice were generated through ENU mutagenesis as previously described [16]. *Hem1^+/−^* mice are phenotypically identical to wildtype mice and hence were used as normal controls. *Rag2^−/−^γ_c_^−/−^* mice were obtained from Taconic Farms, Incorporated (Hudson, NY) and maintained in a breeding colony at the University of Washington. Mice were housed under Specific Pathogen Free conditions. All mouse procedures were performed in accordance with The Guide for the Care and Use of Animals of the National Institutes of Health and approved by the Institutional Animal Care and Use Committee at the University of Washington, which is accredited by the Association for the Assessment and Accreditation of Laboratory Animal Care (AAALAC), International.

### Peripheral Blood Smear Evaluation and Erythrocyte Counting

Peripheral blood smears were stained with differential stain (Dip-Quick, Jorgensen Laboratories, Loveland, CO) and examined at ×600 magnification on a Nikon Eclipse 80i microscope (Nikon, Incorporated, Melville, NY). Images were captured with a Digital Sight DS-U1 camera; adjustments to image size and settings were made using NIS Elements Basic Research 3.0 software (Laboratory Imaging, Limited; Prague, Czechoslovakia). In-house cell counts were conducted using a hemacytometer (Reichert, Buffalo, NY) and a Nikon TMS microscope (Nikon Instruments, Incorporated, Melville, NY) at ×100 magnification.

### Flow Cytometry

Vybrant CFDA SE Cell Tracer Kit carboxyfluorescein succinimidyl ester (CFSE) and Alexa Fluor 488 phalloidin were from Invitrogen (Eugene, OR). Biotin anti-mouse TER-119/Erythroid cells was from Pharmingen (BD Biosciences, San Diego, CA); GR-1 and CD61 antibodies were from eBioscience (San Diego, CA). Erythroblasts (CD11b^−^CD61^−^Ter119^+^) and megakaryocytes CD11b^−^CD61^+^Ter119^−^) were FACs-sorted from total bone marrow for real-time PCR analyses.

For erythrocyte development analyses, flow cytometry was performed on total bone marrow or splenocytes freshly harvested from mice, without lysis of red cells. Phycoerythrin-conjugated rat anti-mouse CD44 was obtained from BD Biosciences (no. 553134) and biotinylated anti-mouse Ter119 was obtained from Pharmingen (no. 090B2D). Cells were stained in DPBS/3% fetal bovine serum (FBS) for 30 min on ice, washed once in DPBS/3% FBS, stained with TRI-COLOR® streptavidin (Invitrogen, no. SA1006) in DPBS/3% FBS for 30 min on ice, washed twice in DPBS/3% FBS and detected using a FACScan™ flow cytometer (BD Biosciences). Live Ter119^+^ cells were gated and separated into developmental stages based on cell size (FSC) and expression of CD44. Data were analyzed using FloJo™ v. 8.6.1 software (Treestar Inc, Ashland OR).

### Purification of Erythrocytes from Whole Blood

Whole blood was obtained from mice immediately following euthanasia via CO_2_ asphyxiation. Red blood cells were purified from whole blood by removal of leukocytes using a cellulose column as previously described by Beutler et al [21]. Briefly, anticoagulated blood (0.5–0.8 mL) collected from CO_2_ euthanized mice was washed in PBS and allowed to pass through a 2-mL column of cellulose. Cellulose columns were composed of equal dry weights α-cellulose and microcrystalline cellulose in 0.154 M NaCl. PBS (10 mM NaCl, 155 mM KCl, 10 mM glucose, 1 mM MgCl_2_, 2.5 mM KHPO_4_, pH 7.4) was allowed to pass through the column after the blood. The first 15 mL of filtrate were spun at 800 *g*×20 min for a total of 3 washes, then lysed as described below.

### Preparation of Erythrocyte Ghosts and Hemoglobin-depleted Cytosolic Fraction

Purified erythrocytes were processed for immunoblot analysis in the presence of MgCl_2_ to protect actin filament capping, as described by Kuhlman and Fowler [4]. Washed erythrocytes were lysed for 30 minutes on ice as per Kalfa et al [3] in NaPi buffer [5 mM sodium phosphate, pH 7.4, 10 mM NaCl, 1 mM EGTA, 2 mM MgCl_2_, with phenylmethylsulfonyl fluoride (PMSF) and sodium orthovanadate (Na_3_VO_4_) added fresh at concentrations of 2 mM and 1 mM, respectively]. After centrifugation, the supernatant (cytosolic fraction) was aspirated and frozen (directly, or in urea-SDS buffer), or depleted of hemoglobin (see “Mass spectrometry [MS] and proteomics”) then frozen (directly, or in urea-SDS buffer). The pellets (membrane ghosts) were then washed at least 3 times in PBS or NaPi buffer and resuspended in urea-SDS sample buffer prior to freezing. All samples were stored at −80°C.

For immunoblotting, samples were solubilized in boiling urea-SDS sample buffer for 5 minutes and loaded according to one of three standards: 1) cell number, 2) total protein, or 3) volume required for equivalent actin loading, as determined by previous immunoblots of the samples for β-actin. Results were similar regardless of the method of standardization.

### Electrophoresis and Western Blotting

SDS-PAGE was performed as previously described by Kalfa et al [3] using a 5% stacking gel and 6–8% running gels. Gels were stained with Coomassie blue or transferred to polyvinylidene fluoride (PVDF) membranes (NEN Life Science Products, Inc., Boston, MA) for immunoblotting. The following antibodies were used: rabbit anti-α-adducin (BioLegend, San Diego, CA, 617002); rabbit anti-ankyrin (kind gift from Raymond Robledo); mouse anti-dematin (Pharmingen/BD Biosciences, San Diego, CA); mouse anti-β1-spectrin (Abcam, Cambridge, MA); rabbit anti-tropomodulin 3 (Lifespan Biosciences, Seattle); mouse anti-phospho-adducin (Millipore Corporation, Temecula); goat anti-WAVE1, goat anti-WAVE2, goat anti-Abi2, and donkey anti-goat IgG HRP from Santa Cruz Biotechnology (Santa Cruz, CA); rabbit anti-Hem1 (kind gift from Orion Weiner); goat anti-rabbit IgG HRP and goat anti-mouse IgG HRP from Bio-Rad Laboratories (Hercules, CA); mouse anti-β-actin (Sigma-Aldrich, St. Louis, MO, A5441).; anti-GYPA, anti-MMP1, and anti-TMOD1 (Sigma-Aldrich, St. Louis Mo. 63103); anti-PP2Ac (BD Biosciences, San Jose, CA 95131), anti-PP2Ar (EMD Chemicals, Gibbstown, NJ 08027).

Developed films were scanned into Adobe Photoshop CS version 8.0 (Adobe Systems Incorporated, San Jose, CA). Scanned images were imported into Canvas version 9.0.2. (ACD Systems International Incorporated, Seattle, WA) for figure preparation.

### Real-time Polymerase Chain Reaction (RT-PCR)

Bone marrow was harvested from *Hem1^−/−^* and age-matched WT or *Hem1^+/−^* mice, and erythroblasts and megakaryocytes were FACs-sorted (FACSAria BD Biosciences, Franklin Lakes, NJ) as previously described [22]. RNA was extracted from erythroblasts and megakaryocytes with the RNAqueous-4PCR kit (Ambion/Applied Biosystems, Austin, TX). cDNA was generated using Superscript II Reverse Transcriptase (Invitrogen, Grand Island, NY). Samples were normalized using *β-actin* [*β-actin* forward (5′ – TCCTTCGTTGCCGGTCCAC –3′) and *β-actin* reverse (3′ – ACCAGCGCAGCGATATCGTC –5′)] primers. *Hem1* levels were determined using *Hem1* forward (5′–AGGTGGCATGCCTACTCTTGATCT –3′) and reverse (5′ – AGAGGCCACCACCAGAAACTCTTT –3′); *Abi1* levels were determined using *Abi1* forward (5′ – TGGCACATTGTCGAGAACAAACCC –3′) and reverse (5′ – ACTGTTGGAGGCTTAACAGGCTCT –3′); *Sra1* levels were determined using *Sra1* forward (5′ – ATGTTCCTGGCCAACCACAACAAG –3′) and reverse (5′ – AGGTACAGGCCAAATCCCATGACT –3′); *WAVE2* levels were determined using *WAVE2* forward (5′ – ACCACCCAAGACCAGAAGCTC –3′) and reverse (5′ – TCCTGCAGCATCTTCTCCTTCCAA). Expression of *Hem1* in erythroblasts relative to β-actin was normalized to relative expression of *Hem1* in murine renal cells, which has previously been shown to be very low [16]. Expression of the WAVE complex genes relative to β-actin was compared to C_T_ value of 38, which represents a low expressing tissue. Experiments were performed using a real-time system sequence detector (PCR GeneAmp PCR System 9700, PE Applied Biosystems, Austin, TX) and PCR system (Mx3005P, Stratagene/Agilent Technologies, Santa Clara, CA) to quantitate the expression of *Hem1*, *Abi1*, *Abi2*, *Sra1*, and *WAVE2* using the comparative C_T_ method [23].

### Erythrocyte Transfusion and Survival

Erythrocytes collected via retro-orbital bleeding of *Hem1^−/−^* and WT mice were purified, washed three times in PBS/0.1% FBS, and were labeled with CFSE per manufacturer’s instructions. 5×10^7^ cells were transfused via retro-orbital injection into *Rag2^−/−^γ_c_^−/−^* host mice under isoflurane anesthesia (*n* = 4 mice receiving WT erythrocytes; *n* = 3 mice receiving *Hem1^−/−^* erythrocytes). Host mice were bled via tail vein at 24 and 48 hours post-transfusion, and at days 5, 9, 16, 23, 28, 36, 42, and 49 post-transfusion. Host erythrocytes were sampled by flow cytometry and data analyzed using FlowJo version 8.6.1 software (Tree Star, Incorporated, Ashland, OR). The percentage of CFSE-labeled erythrocytes in the 24-hour post-transfusion sample was considered 100% labeling, and percentages of labeled erythrocytes at subsequent timepoints were calculated relative to this.

### Immunofluorescence of Erythrocytes

Coverslips for mounting erythrocytes were coated with 50 µL of 0.01% poly-L-lysine and allowed to dry. Peripheral blood was processed as by Kalfa et al [3] with the following modifications: 8 µL of whole blood were fixed with 100–200 µL 0.5% acrolein in solution for 5 min. Washed, diluted erythrocytes were adhered to coverslips for 2 hr, then were permeabilized for 30 min in rinsing solution containing 1.0% Triton X-100. Slides were stained with 0.4 U/slide Alexa Fluor 488 phalloidin (0.2 U/µL solution in methanol, diluted 1∶50 with PBS) and anti-β-spectrin (1∶250 in 3% BSA in PBS) at 4°C overnight, then with goat-anti-mouse secondary antibody labeled with Alexa Fluor 568 (1∶1000 in 3% BSA in PBS; Molecular Probes, Eugene, OR) for 1 hour. Coverslips were mounted with ProLong Gold Antifade (Molecular Probes, Eugene, OR). The spatial distribution of the fluorescent probes was analyzed via confocal laser-scanning microscope (Zeiss LSM 510 META, 1.45 numerical aperture, 1000× magnification).

### Mass Spectrometry (MS) and Proteomics

Wildtype and *Hem1^−/−^* erythrocytes were compared using a label free comparative proteomic approach [24], [25]. Erythrocytes were purified from independent whole blood samples using cellulose chromatography as described above. All subsequent sample processing was standardized so as to minimize pre-analytical variability in the erythrocyte proteome. Erythrocytes were lysed hypotonically and centrifuged at 1000 *g*, 4°C for 15 min. The soluble fraction was removed and the insoluble fraction pellet (erythrocyte ghosts) was washed 5 times in PBS. Samples of soluble fraction samples were hemoglobin-depleted using a modification of the method of Ringrose et al [26]. For each sample, 1.0 mL packed Ni-NTA agarose resin (Qiagen Incorporated, Valencia, CA) was equilibrated by washing three times with 50 mM NaH_2_PO_4_/5 mM imidazole in a 2.0 mL microfuge tube. Hemoglobin-rich supernatant was adjusted to 5 mM imidazole, mixed with the packed Ni-NTA, and incubated with rocking overnight at 4°C. The resin was pelleted by centrifugation for 1 minute at 12,000 rpm at 4°C. The supernatant was combined with 1.0 mL fresh packed Ni-NTA for a second overnight incubation. The resulting clear supernatant was concentrated approximately 80% on a Speed Vac Concentrator (Savant). For all samples, protein concentrations were determined using the BioRad Protein Assay (BioRad Labs, Hercules, CA) and aliquots were stored at −80°C. Protein samples were prepared and profiled using 1 µg of protein per run on a hybrid linear ion trap FTICR mass spectrometer (LTQ-FT Ultra, ThermoElectron, Waltham, MA) as previously described [25]. Peptide and protein identification and label-free analysis were as described previously [25]. Comparisons of the insoluble fraction were performed for retention time regions between 23 and 111 minutes, and utilized a data set of 5 WT technical replicates (derived from 2 biological replicates) and 8 *Hem1^−/−^* technical replicates (derived from 3 biological replicates). Soluble fraction comparisons were performed for retention time regions between 20 and 140 minutes, and utilized a data set of 4 WT technical replicates (derived from 2 biological replicates) and 4 *Hem1^−/−^* technical replicates (derived from 2 biological replicates).

### Statistics

Student’s t-test and Fishers exact test were used to compare means and calculate significance.

## Results

### Hem1-null Erythrocytes Exhibit Altered Morphology and Condensed F-actin Foci

**Figure 1:**
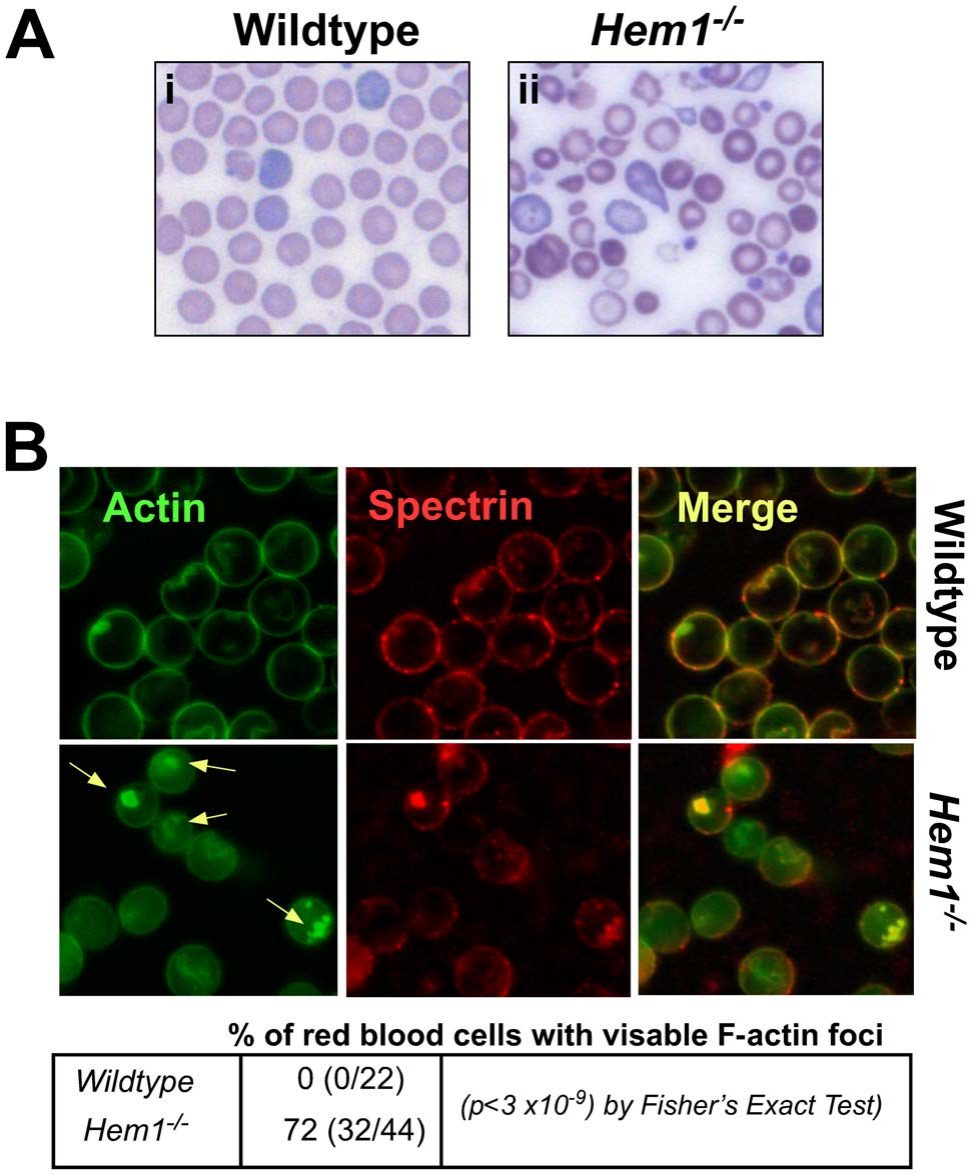
*Hem1^−/−^* erythrocytes are morphologically abnormal and contain condensed F-actin foci. (A) Peripheral blood smears from (i) WT and (ii) *Hem1^−/−^* mice were stained with Wright-Giemsa stain. Shown are representative blood smears (approximately 20 individuals per genotype). Note poikilocytosis, polychromasia, hypochromia, and anisocytosis of *Hem1^−/−^* erythrocytes as well as the increased presence of acanthocytes, schistocytes, dacryocytes, and keratocytes. Original magnification ×600. (B) Confocal microscopy images of WT and *Hem1^−/−^* erythrocytes stained for actin (Actin), β-spectrin (Spectrin), and combined (Merge). Representative of 5 animals per genotype. Note decreased membrane actin and spectrin, and increased areas of aggregated actin in *Hem1^−/−^* versus WT erythrocytes (yellow arrows), many of which co-localize at the periphery with β-spectrin. Most *Hem1^−/−^* erythrocytes (identified by biconcave shape) contain actin foci, as noted through multiple cross sections. No WT erythrocytes were identified with actin foci. A single layer is shown. Alexa Fluor 488 phalloidin stain and anti-β-spectrin labeled with Alexa Fluor 568 are shown, original magnification ×1000.

Our initial analysis of *Hem1^−/−^* mice indicated that loss of Hem1 results in microcytic, hypochromic anemia characterized by abnormal erythrocyte morphology and increased fragility [16]. Detailed characterization of the erythrocyte abnormalities in peripheral blood samples from *Hem1^−/−^* mice using differentially stained blood smears reveal abundant abnormal erythrocytes including acanthocytes, schistocytes, dacryocytes, and keratocytes. *Hem1^−/−^* erythrocytes also exhibit poikilocytosis, anisocytosis, hypochromia, and polychromasia (Figure 1A).

We next sought to determine whether the abnormal erythrocyte morphology in *Hem1^−/−^* mice is associated with aberrant actin polymerization. Using phalloidin and anti-spectrin staining followed by confocal microscopy, we found that whereas actin and spectrin co-localize in the cell membrane of WT erythrocytes, *Hem1^−/−^* erythrocytes contain less membrane-associated actin and spectrin, and increased cytoplasmic actin. *Hem1^−/−^* erythrocytes also contain abnormal foci of brightly staining condensed actin (yellow arrows) relative to WT erythrocytes, which lack condensed actin foci (Figure 1B). Using Deltavision® microscopy, analyses of multiple cross-sections indicate that abnormal actin foci are present in the majority of *Hem1^−/−^* erythrocytes relative to WT erythrocytes (*p*<3×10^−9^) and co-localize with spectrin to the periphery in apparent association with the cell membrane. These studies indicate that disruption of *Hem1* results in altered erythrocyte morphology, which correlates with disrupted organization of the actin membrane skeleton.

**Figure 2:**
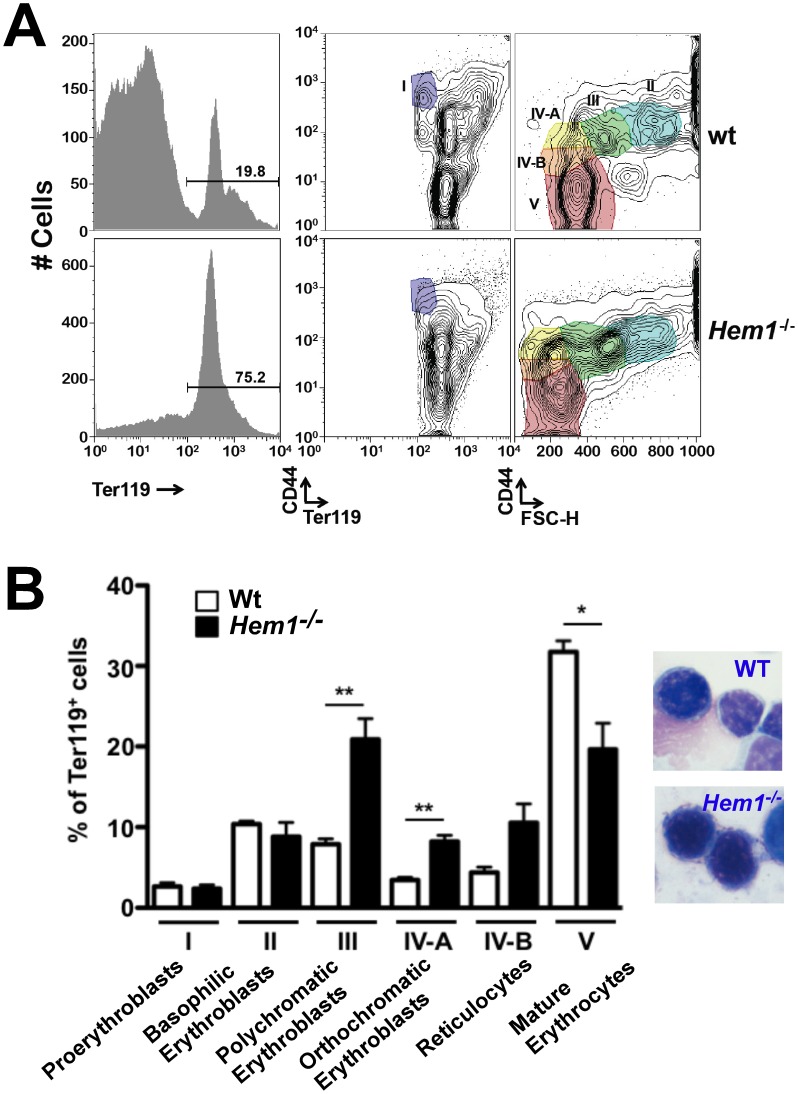
Loss of Hem1 does not impair erythropoiesis. Flow cytometric analyses of splenocytes from *Hem1^−/−^* vs. wildtype (wt) littermate control mice. (A) Live-gated, Ter119^+^ cells (left panels) are shown plotted by cell-surface display of CD44 vs. Ter119^+^ (middle panels) or CD44 vs forward scatter (right panels). Gates defining different erythroid progenitor populations (I-V) are shown. (B) (*left*) Erythroid progenitor populations are shown as percent of Ter119^+^ cells. Bars represent mean +/− SEM for three animals per genotype. The asterisks indicate statistical significance: (*), *p*<0.01; (**), *p*<0.001. (*right*) *Hem1^−/−^* erythroblasts have normal morphology. Shown are representative photos of WT and *Hem1^−/−^* erythroblasts.

We next sought to determine whether the anemia in *Hem1* ^−/−^ mice is the result of abnormal erythropoiesis and/or the removal of abnormally shaped mature erythrocytes as they circulate in peripheral blood. To assess erythropoiesis in *Hem1^−/−^* and WT mice, we identified the percentage and number (not shown) of erythrocytes in different stages of erythrocyte development based on the expression of the CD44 adhesion molecule on Ter119^+^ erythroid lineage cells, in combination with forward light scatter characteristics [27]. We found that while there is less erythropoiesis in *Hem1^−/−^* bone marrow due to increased myelopoiesis and changes in the microenvironment as previously shown [16], splenic erythropoiesis is significantly increased in *Hem1^−/−^* mice relative to WT mice based on increased percentage and number (not shown) of total Ter119^+^ erythroid lineage cells (Figure 2A, left), and increased percentage of polychromatic erythroblasts (Stage III), orthochromatic erythroblasts (Stage IV-A), and reticulocytes (Stage IV-B) (Figure 2B, left) ( [16]). *Hem1^−/−^* erythroblasts also have normal membrane structure relative to WT erythroblasts (Figure 2B, right). In contrast, the morphology of mature erythrocytes is significantly altered (Figure 1A) and the percentage of mature *Hem1^−/−^* erythrocytes (Stage V) is selectively and significantly decreased indicating that anemia in *Hem1^−/−^* mice likely results from the loss of abnormally shaped mature erythrocytes and not from cell intrinsic impairment in erythrocyte development. These findings are also consistent with our previous demonstration that *Hem1^−/−^* erythrocytes are more fragile relative to WT erythrocytes in response to osmotic stress [16].

### Hem-1 and WAVE Complex Components are Expressed in Erythrocytes and Erythrocyte Progenitors

**Figure 3:**
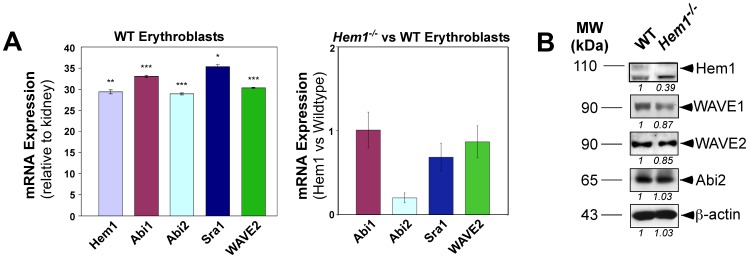
Expression of WAVE complex mRNA and protein in wildtype (WT) and *Hem1^−/−^* mouse erythrocytes. (A) (*Left*) Real-time PCR of FAC-sorted erythroblasts from WT mice shows relative expression of *Hem1* and WAVE complex (*Abi1, Abi2, Sra1*, and *Wave2*) mRNA normalized to *β-actin*. Expression of *Hem1* mRNA is shown relative to kidney mRNA. Expression of WAVE component mRNAs are shown relative to a C_T_ value of 38, which represents an arbitrary low expressing tissue. (*), *p*<0.0007; (**), *p*<0.0004; (***), *p*<0.0001. *n* = 3 mice per genotype. (*Right*) Expression of WAVE complex mRNA are shown in Hem1 erythroblasts versus WT erythroblasts. *Abi2 p<0.0004* (B) Immunoblots of purified erythrocyte ghosts from WT and *Hem1^−/−^* mice, loaded according to equivalent levels of β-actin. Each lane is representative of at least 3 individuals per genotype. Hem-1 protein (upper band, 110 kDa) is not detectable in erythrocyte ghosts from *Hem1^−/−^* mice. Although *Abi2* mRNA levels are reduced in *Hem1^−/−^* erythrocytes, Abi2 protein levels are relatively normal, likely due to post-transcriptional and/or post-translational regulation. Numbers below each scan represent protein expression levels in *Hem1^−/−^* relative to WT samples. Samples were loaded based on equivalent total protein.

We had previously shown that *Hem1* is expressed predominantly in hematopoietic cells including B cells, T cells, macrophages, and neutrophils [16]. Since *Hem1^−/−^* mice also present with defects in erythrocyte biology, we examined whether *Hem1* and *WAVE* complex mRNA and protein are also expressed in erythrocytes and erythrocyte progenitors. Since mature erythrocytes lack nuclei, we first examined the expression of *Hem1* and *WAVE* complex mRNA by real-time PCR on samples derived from FACS-sorted erythroblasts from WT and *Hem1^−/−^* mice. WT erythroblasts expressed high levels of mRNA encoding components of the WAVE complex including *Hem1, Abi1, Abi2, Sra1,* and *WAVE2* (Figure 3A, left). *Hem1^−/−^* erythroblasts expressed equivalent levels of *Abi1, Sra1,* and *WAVE2* mRNA, whereas *Hem1* (not shown) and *Abi2* were significantly decreased (Figure 3A, right). Immunoblots of purified mature erythrocyte lysates from WT and *Hem1^−/−^* mice also indicated that WAVE proteins including Abi-2, WAVE-1, and WAVE-2 are present in nearly equal amounts relative to β-actin, whereas Hem-1 protein (top band) is completely absent (Figure 3B). This is in contrast to Hem-1 null lymphocytes and neutrophils, where WAVE complex proteins were significantly less abundant relative to WT cells [16]. These results suggest that Hem-1 and members of the WAVE complex are expressed in erythrocytes and that individual protein components of the WAVE complex are relatively stable in erythrocytes, even in the absence of Hem-1.

### Global Alterations in Protein Expression in *Hem1^−/−^* Erythrocytes

**Figure 4:**
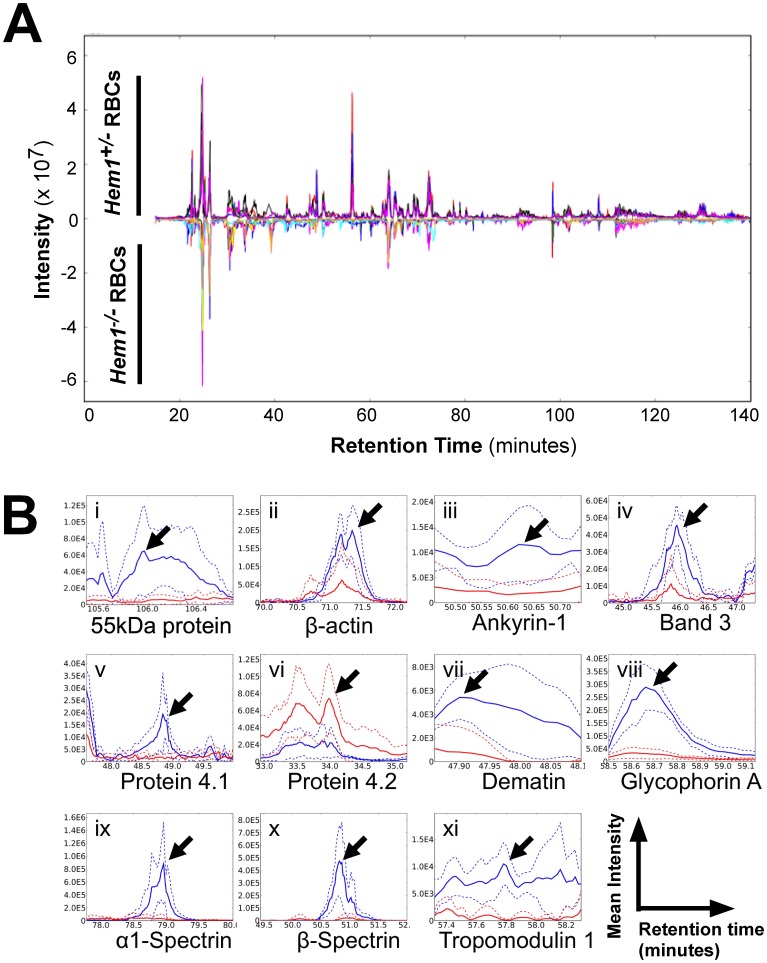
WT and *Hem1^−/−^* erythrocytes show differential expression of key membrane and structural proteins as revealed by proteomics. (A) Chromatographic alignments of replicate runs for WT and *Hem1^−/−^* erythrocytes. Alignments of 5 WT (positive inflection line) and 8 *Hem1^−/−^* (negative inflection line) erythrocyte technical replicate µLC-MS runs are shown in a base peak plot (ion intensity vs. retention time). Individual replicates are shown in distinct colors for each genotype. (B) CRAWDAD detection of chromatographic difference regions for 55 kDa erythrocyte membrane protein peptide TAELSPFIVFIAPTDQGTQTEALQQLQK (i), actin peptide SYELPDGQVITIGNER (ii), ankyrin 1 peptide AGKEPSLWAPESA (iii), band 3 peptide YLPSPAKPDPNLYN (iv), band 4.1 peptide LIDRPAPHFER (v), band 4.2 peptide AAQYRPLTVSVR (vi), dematin peptide STSPPPSPEVWAESR (vii), glycophorin A peptide GDNSVPLSSIEQTPNEESSNV (viii), spectrin alpha peptide AEQVDGVINLGNSLIER (ix), spectrin beta peptide FAALEKPTTLELK (x), and tropomodulin 1 peptide LADLTGPIIPK (xi). Aligned replicate µLC-MS runs from the WT and *Hem1* ^−/−^ erythrocyte series are shown in blue and red, respectively. Mean intensity values are shown in solid lines and ±1 SD in dashed lines. Difference regions for each peptide indicated with arrows.

We next utilized an unbiased global proteomics approach to investigate why loss of Hem-1 results in anemia and defects in the actin cytoskeleton. Specifically, we compared the abundance of a broad range of proteins present in WT and *Hem1* deficient erythrocytes using the label-free CRAWDAD (Chromatographic Retention Time Alignment and Warping for Differential Analysis of LC-MS Data) mass spectrometry approach [24], which uses information in LC-MS/MS spectra to identify the peptides present in a protease digest to estimate differences in abundance of peptides from their intensities in LC-MS spectra. Using this approach, we identified statistically significant differences in the abundance of 14 proteins in the insoluble (membrane bound) fraction (Figures 4A, 4B and Table 1) and 74 proteins in the soluble (cytosolic) fraction (Table 2) between *Hem1^−/−^* and WT erythrocytes. A complete list of proteins identified in these samples is presented in supplementary data (Tables S1–S4), and a complete list of peptide differences detected in the insoluble and soluble fractions is presented in Tables S5 and S6. Examples of peptide differences detected between WT and *Hem1^−/−^* insoluble fraction samples by CRAWDAD are illustrated in Figure 4B. The majority of the significant changes were associated with decreased protein levels in *Hem1^−/−^* erythrocytes relative to WT erythrocytes. Notably, important cytoskeletal proteins including protein 4.1, tropomodulin 1, dematin, band 3, β-actin, p55, α- and β- spectrin, and ankyrin were significantly decreased in the insoluble fractions from *Hem1^−/−^* erythrocytes relative to WT erythrocytes (Figure 4B and Table 1), consistent with the profound changes in erythrocyte morphology and actin foci noted by microscopy. Interestingly, the soluble cytosolic fractions from *Hem1^−/−^* erythrocytes contained significantly decreased representation of a number of essential metabolic enzymes associated with glycolysis [such as glyceraldehyde-3-phosphate dehydrogenase (GAPDH), phosphoglycerate kinase, and β- and γ- enolase] and other biochemical pathways (including malate dehydrogenase, phosphoglycolate phosphatase, phosphoglucomutase-2, fumarate hydratase, and 6-phosphogluconolactonase) relative to WT erythrocytes (Table 2). We also found significantly decreased levels of enzymes directly or indirectly responsible for inactivation of reactive oxygen species (ROS), such as phospholipid hydroperoxide glutathione peroxidase, and enzymes involved in protein repair (including ketosamine-3-kinase) and oxygen exchange, such as bisphosphoglycerate mutase. Importantly, and as further discussed below, Hem-1 null erythrocytes contained significantly decreased amounts of the regulatory A subunit (−7.06 fold) and regulatory B subunit (−10.16 fold) of the serine-threonine protein phosphatase 2A (PP2A) [28], which dephosphorylates proteins that are typically phosphorylated by protein kinase C (PKC) [29], including adducin.

**Table 1:** Protein differences identified in insoluble fraction via proteomics in WT and *Hem1^−/−^* erythrocytes.

Protein		Intensity Difference*	No. of Peptides	ID
1	Hemoglobin, alpha	−27.38	7	IPI00845802.1
2	β-Spectrin	−15.99	7	IPI00131376.5
3	Ankyrin 2	−14.96	2	IPI00227235.4
4	Ankyrin 3	−14.96	2	IPI00173248.2
5	α-Spectrin	−11.46	20	IPI00896567.1
6	Protein band 4.1	−11.41	5	IPI00649005.1
7	Tropomodulin 1	−11.01	2	IPI00655150.1
8	Glycophorin A	−8.81	3	IPI00123673.1
9	Actin, beta	−5.28	5	IPI00110850.1
10	Dematin	−5.09	3	IPI00125328.3
11	55 kDa erythrocyte membrane protein	−4.87	2	IPI00137706.1
12	Ankyrin 1	−4.85	15	IPI00119871.3
13	Protein band 3 anion transport protein	−3.30	16	IPI00120761.3
14	Erythrocyte protein band 4.2	2.62	2	IPI00421166.3

*n* = 2 WT mice (5 technical replicates) and 3 *Hem1^−/−^* mice (8 technical replicates).

* Fold difference in *Hem1^−/−^* versus WT erythrocytes. *p*≤0.05.

**Table 2:** Protein differences identified in soluble fractions via proteomics in *Hem1^−/−^* versus Wt erythrocytes.

Protein		Intensity Difference*	No. of Peptides	ID
1	Parkinson disease (Autosomal recessive, early onset) 7	−155.1	3	IPI00125923.1
2	Osteoclast-stimulating factor 1	−86.32	2	IPI00336324.1
3	Malate dehydrogenase, cytoplasmic	−32.12	5	IPI00137758.5
4	Secernin-3	−31.47	3	IPI00649794.1
5	100042424 Programmed cell death protein 5	−26.07	2	IPI00116120.3
6	Vinculin	−25.24	2	IPI00405227.3
7	Glia maturation factor, β	−21.53	2	IPI00467495.4
8	β-enolase	−21.15	4	IPI00228548.6
9	Glucosamine-6-phosphate isomerase 1	−20.98	2	IPI00379245.2
10	Secernin 3, isoform CRA_b	−20.32	2	IPI00756194.1
11	Isoform Er1 of Ankyrin-1	−18.13	5	IPI00127163.4
12	Eukaryotic translation initiation factor 6	−16.62	3	IPI00857575.2
13	14-3-3 protein epsilon	−16.41	2	IPI00118384.1
14	Dipeptidyl-peptidase 3	−11.52	2	IPI00116134.1
15	γ-enolase	−11.17	2	IPI00331704.7
16	Serine/threonine-protein phosphatase 2A regulatory subunit B’	−10.16	4	IPI00118723.3
17	Nucleosome assembly protein 1-like 4	−9.71	2	IPI00876202.1
18	UMP-CMP kinase	−9.59	2	IPI00331146.5
19	Spectrin α chain, erythrocyte	−9.58	5	IPI00323230.5
20	Phosphoglycerate kinase 1	−9.23	8	IPI00555069.3
21	Phosphoglycolate phosphatase	−8.65	2	IPI00380195.1
22	Ubiquitin-conjugating enzyme E2 N	−7.86	2	IPI00165854.3
23	Adenosylhomocysteinase	−7.7	2	IPI00230440.6
24	Phospholipid hydroperoxide glutathione peroxidase	−7.6	2	IPI00463220.2
25	Erythrocyte membrane protein band 4.2	−7.47	2	IPI00421166.3
26	Serine/threonine-prot. phosphatase 2A 65 kDa regulatory, subunit A α isoform	−7.06	2	IPI00310091.8
27	26S protease regulatory subunit 6A	−7.03	4	IPI00133206.1
28	Proteasome subunit β type-4	−7	3	IPI00129512.3
29	Heat shock protein HSP 90-α	−6.92	2	IPI00330804.4
30	Putative uncharacterized protein	−6.76	2	IPI00458922.1
31	Ketosamine-3-kinase	−6.68	2	IPI00321994.1
32	Myotrophin	−6.65	2	IPI00228583.5
33	Heat shock protein 2	−6.35	2	IPI00387494.1
34	Ribonuclease inhibitor	−6.35	3	IPI00313296.3
35	OTTMUSG00000014946 hypothetical protein	−6.22	6	IPI00762304.1
36	V-type proton ATPase subunit B, brain isoform	−6.03	3	IPI00119113.3
37	Moesin	−5.96	3	IPI00110588.4
38	Bisphosphoglycerate mutase	−5.96	3	IPI00221663.5
39	Glutamate–cysteine ligase regulatory subunit	−5.77	2	IPI00114329.1
40	Peroxiredoxin 6, related sequence 1	−5.71	2	IPI00221454.1
41	LOC100048075 similar to nonselenium glutathione peroxidase	−5.58	4	IPI00756385.1
42	Peroxiredoxin-6	−5.57	5	IPI00555059.2
43	36 kDa protein	−5.56	8	IPI00399459.2
44	Glyceraldehyde-3-phosphate dehydrogenase	−5.55	10	IPI00273646.9
45	Inorganic pyrophosphatase	−5.54	2	IPI00110684.1
46	14-3-3 protein ζ/δ	−5.31	2	IPI00116498.1
47	Proteasome subunit alpha type-7/Psma8 Proteasome subunit a-type-7-like	−5.3	2	IPI00131406.1
48	Similar to Proteasome 26S subunit, non-ATPase, 2 isoform 1	−5.25	2	IPI00675436.2
49	26S proteasome non-ATPase regulatory subunit 2	−5.25	2	IPI00123494.3
50	Isoform Erythrocyte of Band 3 anion transport protein	−5.23	5	IPI00120761.3
51	GTP-binding nuclear protein Ran, testis-specific isoform	−4.99	2	IPI00126133.1
52	Phosphoglucomutase-2	−4.96	2	IPI00338302.1
53	Isoform 1 of Cytosolic 5′-nucleotidase 3	−4.96	2	IPI00648235.1
54	Spectrin β1	−4.72	4	IPI00131376.5
55	Fumarate hydratase, mitochondrial	−4.71	2	IPI00129928.2
56	Lactoylglutathione lyase	−4.56	6	IPI00321734.7
57	Proteasome subunit α type-4	−4.56	2	IPI00277001.4
58	LOC100046081 Ubiquitin thioesterase OTUB1	−4.52	2	IPI00154004.1
59	Cofilin-1	−4.48	4	IPI00890117.1
60	6-phosphogluconolactonase	−3.97	2	IPI00132080.1
61	Nuclear transport factor 2	−3.92	3	IPI00124149.1
62	Aldose reductase	−3.89	2	IPI00223757.4
63	36 kDa protein	−3.39	6	IPI00625893.3
64	Nans Putative uncharacterized protein	−3.17	2	IPI00114925.1
65	Eukaryotic translation initiation factor 5A-2	−3.17	2	IPI00331514.6
66	1700009N14Rik RIKEN cDNA 1700009N14 gene	−2.69	2	IPI00127109.4
67	Actin, cytoplasmic	34.35	3	IPI00110850.1
68	Serine/threonine-protein phosphatase 2A catalytic subunit β isoform	27.16	2	IPI00111556.1
69	Serine/threonine-protein phosphatase 2A catalytic subunit α isoform	25.19	2	IPI00120374.1
70	Gart phosphoribosylglycinamide formyltransferase	8.32	2	IPI00230612.3
71	Sepiapterin reductase	7.83	3	IPI00129164.1
72	Isoform 1 of Glyoxalas domain-containing protein 4	6.47	2	IPI00110721.5
73	Putative uncharacterized protein	4.53	2	IPI00830250.1
74	22 kDa protein	2.96	2	IPI00605090.5

*n* = 2 WT mice (4 technical replicates) and 2 *Hem1^−/−^* mice (4 technical replicates).

* Fold difference in *Hem1^−/−^* vs WT erythrocytes. *p = *0.05.

### *Hem1^−/−^* Erythrocytes Exhibit Altered Representation of Junctional and Actin-binding Membrane Skeletal Proteins

**Figure 5:**
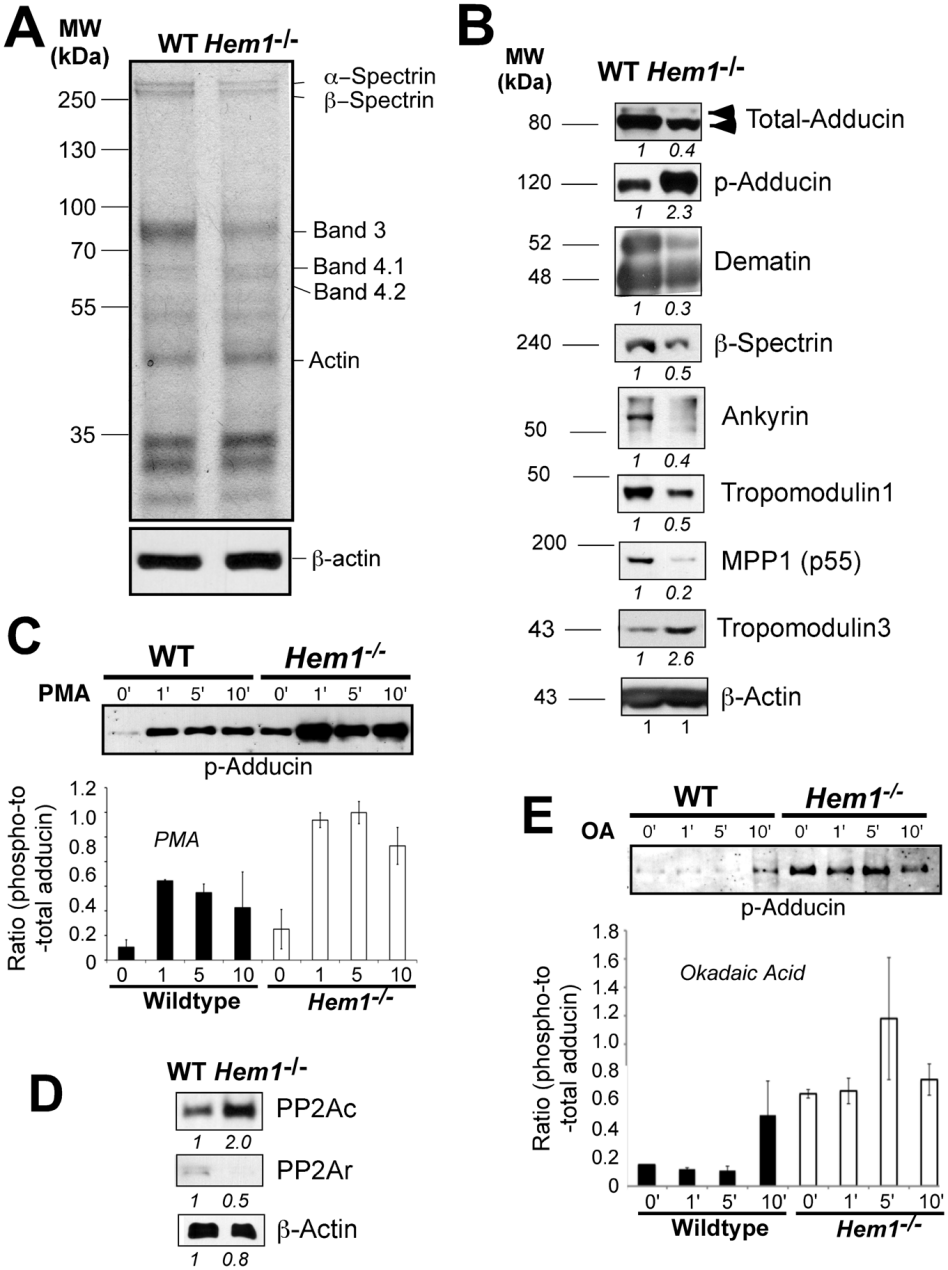
*Hem1^−/−^* erythrocytes contain increased phospho-adducin and decreased levels of essential membrane skeletal proteins. SDS-PAGE analysis of purified erythrocyte ghosts was used to evaluate relative levels of erythrocyte membrane skeletal proteins between WT and *Hem1^−/−^* mice. (A) Coomassie-stained polyacrylamide gel (representative of 6 animals per genotype). Lanes contain equivalent amounts of total protein. β-actin immunoblot is shown below. (B) Immunoblots of erythrocyte membrane ghosts from purified WT and *Hem1^−/−^* erythrocytes, loaded according to total protein and equivalent β-actin. Each pair is representative of results for at least 5 individuals of each genotype. (C) Activation of PKC results in increased phosphorylation of adducin on Serine 724. Purified WT and *Hem1^−/−^* erythrocytes were stimulated with the phorbol ester PMA (20 ng/ml) and were harvested and lysed at the indicated timepoints post-stimulation. A representative immunoblot of three separate experiments is shown (3 animals per genotype). (D) Erythrocyte ghosts from *Hem1^−/−^* mice contain increased PP2A catalytic subunit (PP2Ac) and decreased PP2A regulatory subunit (PP2Ar) protein relative to WT erythrocytes. Shown are total protein levels determined by immunoblot. Numbers below each scan represent relative protein expression levels in *Hem1^−/−^* versus WT samples. Samples were loaded according to levels of total protein. (E) WT and *Hem1^−/−^* erythroblasts were stimulated in Okadaic acid (OA) and were harvested and lysed at the indicated timepoints post-stimulation. A representative immunoblot of 3 separate experiments is shown. Samples were loaded based on equivalent cell number.

Since mass spectrometric analyses indicated that multiple critical membrane skeletal proteins were decreased in *Hem1^−/−^* erythrocytes, we further examined the representation of prominent membrane skeletal proteins in *Hem1^−/−^* erythrocytes by SDS-PAGE and immunoblotting. We first utilized Coomassie-stained gels to analyze the representation of the most abundant non-hemoglobin erythrocyte proteins. We found that *Hem1^−/−^* erythrocytes had decreased levels of band 4.1, band 3, and α- and β- spectrin compared to WT erythrocytes (Figure 5A), which was highly consistent with our proteomics results. *Hem1^−/−^* erythrocyte membrane ghosts (membrane fractions) also exhibited decreased levels of adducin, dematin, β-spectrin, ankyrin, tropomodulin1, and p55 (MPP1) by immunoblot (Figure 5B). Interestingly, both phospho-adducin (which targets adducin for dissociation from actin and degradation) and tropomodulin 3 were increased in *Hem1^−/−^* erythrocytes relative to WT erythrocytes.

In neurons and platelets, adducin is serine-phosphorylated by protein kinase C (PKC) [30], [31] and PKC-mediated phosphorylation is typically opposed by protein phosphatase 2A (PP2A) mediated dephosphorylation. To determine whether adducin is also phosphorylated by PKC in erythrocytes, we treated WT and *Hem1^−/−^* erythrocytes with the PKC-activator phorbol 12-myristate 13-acetate (PMA) for increasing amounts of time. We found that both WT and *Hem1^−/−^* erythrocytes are phosphorylated by PKC on serine 724 and that “basal” serine 724 phosphorylation is greater in the absence of Hem-1 (Figure 5C). Following the addition of PMA, adducin is phosphorylated to a greater extent in the absence of Hem-1. To determine why Hem-1 null erythrocytes exhibit increased serine phosphorylation, we assessed levels of the regulatory (PP2Ar) and catalytic (PP2Ac) subunits of the PP2A phosphatase in ghost fractions from *Hem1^−/−^* and WT erythrocytes by immunoblotting. We found that PP2Ar, which regulates PP2A substrate specificity and catalytic activity, is decreased and PP2Ac is increased in *Hem1^−/−^* versus WT erythrocytes. These results are highly consistent with our proteomics data which also indicated that loss of *Hem1* results in significantly reduced expression of PP2Ar A and B subunits and increased expression of the PP2Ac α and β subunits (Table S1).

### *Hem1^−/−^* Erythrocytes have Decreased Lifespan

**Figure 6:**
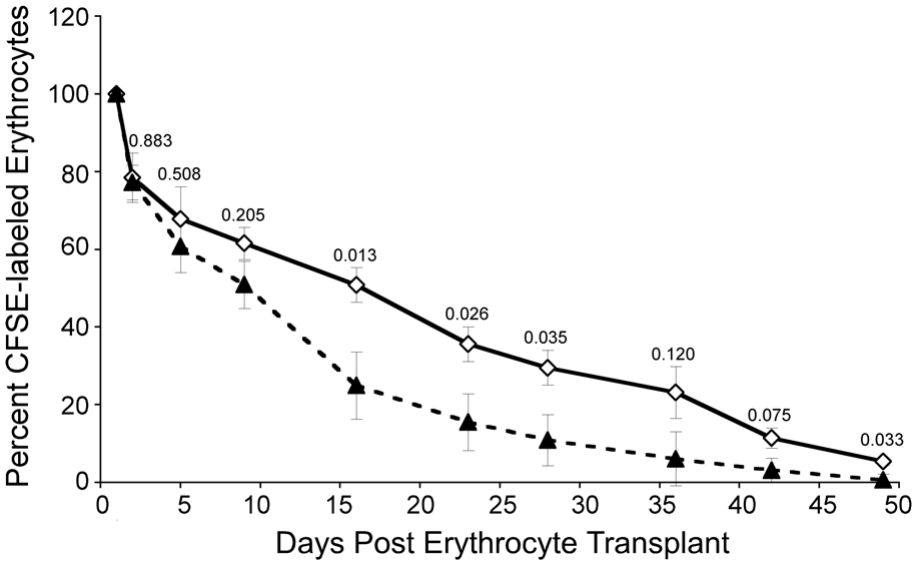
Lifespan of transfused *Hem1^−/−^* erythrocytes is decreased compared to transfused WT erythrocytes. Purified WT (open diamonds, *n* = 4 mice) and *Hem1^−/−^* (filled triangles, *n* = 3 mice) erythrocytes (5×10^8^ per mouse) were labeled with CFSE dye and transfused via retro-orbital injection into *Rag2^−/−^γ_c_^−/−^* host mice. Host mice were tail bled starting Day 1 post-transfusion and samples were analyzed via flow cytometry to determine the percentage of CFSE-labeled erythrocytes. Percent CFSE labeled cells remaining post transfusion were determined relative to Day 1 (100% labeling). *P*-values are noted above each time-point.

Defects in primary structural proteins have been shown to result in abbreviated erythrocyte survival due to decreased deformability, increased splenic trapping, and hemolysis [7]. To determine whether Hem-1 null mice have reduced numbers of erythrocytes due to shortened erythrocyte survival, we measured the lifespan of *Hem1^−/−^* erythrocytes relative to WT erythrocytes following transplantation into identical host environments. Erythrocytes were labeled with CSFE dye and adoptively transferred into immunodeficient *Rag2^−/−^γ_c_^−/−^* mice [32], which lack T, B, and NK lymphocytes and thus eliminate any potential differences in the susceptibility to immune rejection. The percentage of CFSE-positive erythrocytes present in the hosts was then measured at approximately weekly intervals until 50 days post-transfusion. We found that the percentage of *Hem1^−/−^* erythrocytes began declining at a faster rate than WT erythrocytes by Day 5 post-transfusion (Figure 6). From day 15 post-transfusion until the end of the study 50 days post-transfusion, the percentage of circulating *Hem1^−/−^* erythrocytes remained less than half that of WT erythrocytes. These results indicate that *Hem1^−/−^* erythrocytes have significantly reduced lifespans relative to WT erythrocytes, and that the defects causing the abbreviated *Hem1^−/−^* erythrocyte survival are cell-autonomous.

## Discussion

The majority of erythrocyte disorders in humans are associated with mutations in genes encoding important structural proteins, which result in disruption of the erythrocyte actin membrane skeleton, decreased deformability, and subsequent removal of erythrocytes from circulation [1]. In this study, we show for the first time that the actin-regulatory protein Hem-1 and the associated WAVE complex are expressed in erythrocytes and erythrocyte progenitors. In addition, we find that the deletion of *Hem1* in mice results in cell-autonomous microcytic, hypochromic anemia characterized by malformed erythrocytes with abnormal condensed F-actin foci, decreased representation and altered stoichiometry of essential membrane cytoskeletal proteins, and disrupted “metabolon”. These results collectively indicate that Hem-1 has previously unrecognized biochemical role(s) in regulating the formation and/or stability of the actin membrane skeleton in erythrocytes.

Orthologs of Hem-1 have been shown to be components of the WAVE complex, which signals downstream of Rac to stimulate F-actin polymerization (see [18] for review). In immune cells such as lymphocytes and neutrophils, disruption of Hem-1 results in destabilization and degradation of the WAVE complex [16], [33], which prevents WAVE from stimulating ARP2/3-mediated nucleation of F-actin [19], [34]. Other studies on WAVE orthologs have also shown that knockdown or ablation of any protein in the WAVE complex (including Hem-1/2) induces instability and degradation of the remaining WAVE complex protein subunits [19], [34]. However, despite abnormal F-actin organization in mature *Hem1^−/−^* erythrocytes, we found nearly equal amounts of WAVE-1, WAVE-2, and Abi-2 proteins in lysates from mature *Hem1^−/−^* erythrocytes relative to WT erythrocytes, indicating that loss of Hem-1 does not significantly impair stability of the WAVE complex in erythrocytes. We also did not find upregulation of *Hem2* in *Hem1^−/−^* erythroblasts, suggesting that Hem-2 does not compensate for the absence of Hem-1 in erythrocytes (data not shown). These results suggest that differences in the stability of WAVE complex proteins in immune cells and erythrocytes may reflect differences in active inducible actin polymerization in immune cells versus constitutive actin polymerization in erythrocytes. In immune cells, actin polymerization is inducible in response to activation by antigens or pathogen-associated molecular patterns (PAMPs), which assists in limiting the generation of actin-based structures (such as podosomes and phagosomes, and the activation of adhesion) for situations requiring protection from pathogens, thus protecting against excessive inflammation and autoimmunity [12], [35], [36]. In contrast, erythrocytes may not have evolved mechanisms to degrade the WAVE complex acutely since actin polymerization in erythrocytes is constitutive.

To investigate how loss of Hem-1 results in disruption of the actin membrane skeleton in erythrocytes, we utilized an unbiased global proteomics approach to compare the relative representation of specific proteins in erythrocytes from *Hem1^−/−^* and WT mice. We noted statistically significant decreases in the representation of essential membrane and membrane cytoskeletal proteins including α- and β- spectrin, ankyrin, dematin, tropomodulin 1, protein 4.1, and band 3 in *Hem1^−/−^* erythrocytes. We further confirmed by Coomassie staining that *Hem1^−/−^* erythrocytes contain significantly decreased levels of α-spectrin, β-spectrin, band 4.1, and band 3 relative to WT erythrocytes. Immunoblot analyses of erythrocyte ghosts confirmed that β-spectrin, total-adducin, dematin, ankyrin, tropomodulin 1, and MPP1 (p55) were decreased relative to actin in *Hem1^−/−^* erythrocytes. These results collectively suggest that the absence of Hem-1 in erythrocytes results in altered homeostasis of essential membrane and membrane skeletal proteins, which results in defects in cytoskeletal structure, stability, deformability, and likely the localization of key biochemical events such as glycolysis [37].

**Figure 7:**
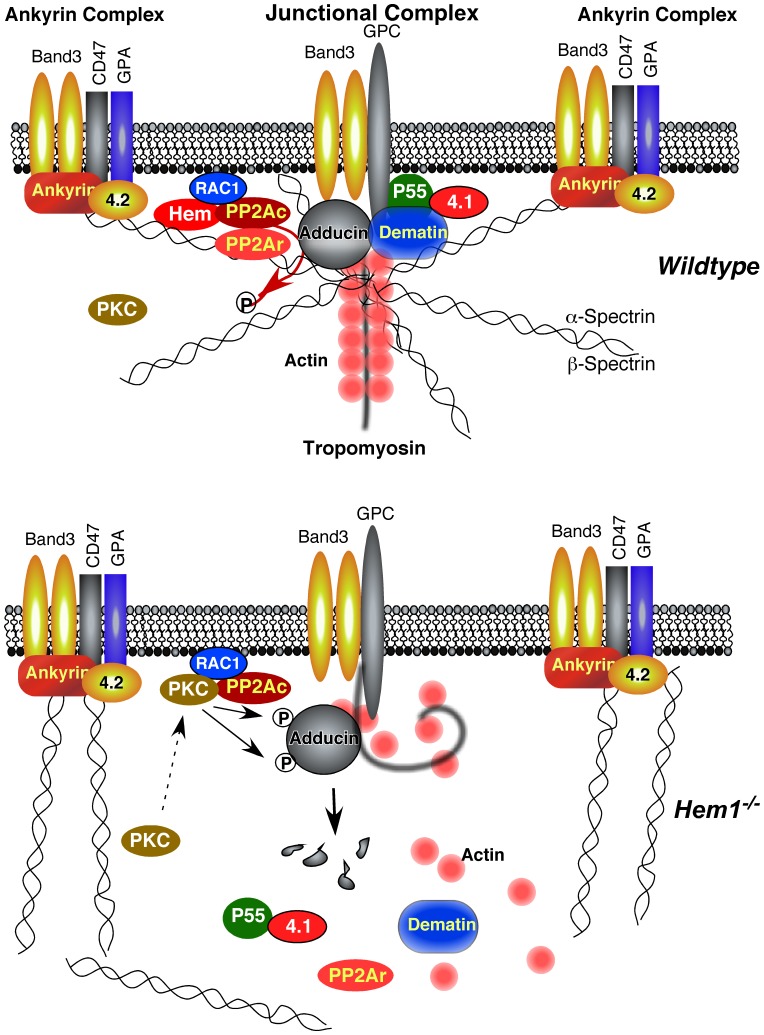
Model of the erythrocyte membrane cytoskeleton in wildtype and Hem-1 null mice. (*top*) The red cell membrane in WT mice consists of a lipid bilayer embedded with two main complexes of structural proteins: The ankyrin complex and the junctional complex (also known as the 4.1R complex), which are connected by horizontal flexible helices of α- and β- spectrin heterodimers and tetramers. Stability of the complexes is regulated in part by phosphorylation of adducin (on Serine 724 in mice) by protein kinase C (PKC), which leads to decreased F-actin capping and dissociation of spectrin from actin. Since PKC-mediated serine phosphorylation is typically opposed by protein phosphatase 2A (PP2A), we propose that PP2A dephosphorylates adducin. PP2A, PKC (bottom), and Hem-1 (top) have all been shown to associate with Rac1. Loss of Hem-1 results in decreased PP2a regulatory subunit B (PP2Ar) and structural subunit A protein expression and increased PKC-mediated phosphorylation of Ser724 on adducin (bottom). Phospho-adducin is then degraded, resulting in the dissociation of spectrin from actin and decreased stability of junctional complex proteins and the membrane cytoskeleton. GPA (Glycophorin A), GPC (Glycophorin C), PP2Ac (protein phosphatase 2A catalytic subunit), PP2Ar (protein phosphatase 2A regulatory subunit).

Interestingly, we also found via immunoblot that phosphorylation of α-adducin at Ser724 was increased in *Hem1^−/−^* erythrocytes, as were the relative levels of tropomodulin 3. In erythrocytes, adducin caps (stabilizes) actin protofilaments at the rapidly-growing barbed ends, while tropomodulin caps actin at the slow-growing pointed ends. In human platelets, phosphorylation of adducin on Ser726 (corresponding to Ser724 on mouse α-adducin) causes dissociation of adducin from spectrin and actin [31], resulting in proteolysis of adducin by calpain [38]. Similarly, in renal epithelial cells phosphorylated α-adducin is cleaved by caspase, which results in its dissociation from spectrin-actin [39]. In neurons and platelets, adducin is serine-phosphorylated by protein kinase C (PKC) [30], [31] resulting in decreased F-actin capping. In *Hem1^−/−^* erythrocytes, we found increased phospho-adducin (Ser724), which correlated with altered representation of membrane skeletal proteins and abnormal condensation of F-actin into discrete foci. We also found that adducin is phosphorylated at Ser724 by PKC in erythrocytes, and that adducin was hyper-phosphorylated in the absence of Hem1. We further determined by mass spectrometry that *Hem1* null erythrocytes contain significantly reduced levels of the protein phosphatase 2A (PP2A) regulatory subunits A (−7.06 fold) and B (−9.58 fold) which are required for PP2A function, and increased levels of PP2A catalytic subunit isoforms α (25.19 fold) and β (27.16 fold), which are known to be upregulated in response to reduced PP2A function [40]. In addition, we confirmed by immunoblot that PP2Ar is reduced and PP2Ac is increased in *Hem1^−/−^* erythrocytes. PP2A is the major phosphatase that dephosphorylates and opposes PKC-mediated serine/threonine phosphorylation. Hence, our studies suggest that disruption of Hem-1 in erythrocytes results in an imbalance between PP2A regulatory and catalytic subunits (which is known to result in reduced PP2A function [41]) and increased phosphorylation of adducin on Ser724. Increased phosphorylation of adducin leads to further dissociation of actin from spectrin and adducin, and overall alterations in membrane skeletal organization and stability. The mechanism of how disruption of Hem-1 results in reduced expression of PP2A regulatory subunits requires further investigation. However, Hem-1 [33], PKC [42], and PP2A [43] have all been shown to interact with Rac, suggesting that disruption of Hem-1 could alter the stoichiometry of these enzymes and shift the balance towards increased phospho-adducin (Figure 7).

In addition to the spectrins, actin, and adducin, we observed decreased levels of other erythrocyte junctional complex proteins including dematin [44], tropomodulin 1 (Tmod1) [45], p55 [46], and protein 4.1 in lysates from *Hem1^−/−^* erythrocytes relative to WT erythrocytes. The junctional complex creates a bridge between the membrane phospholipid bilayer and the underlying cytoskeleton, and many previous studies have shown that this complex is essential for the maintenance of erythrocyte shape, membrane stability, and mechanical properties [44]. For example, Tmod1-null mice exhibit anemia associated with increased osmotic fragility and reduced red blood cell deformability [10]. Expression of tropomodulin 3 (which does not normally occur in wildtype erythrocytes) is upregulated in Tmod1-null erythrocytes perhaps as a compensatory mechanism to aid in capping actin protofilaments. We also observed increased tropomodulin 3 expression in *Hem1^−/−^* erythrocytes. Similarly, mice deficient in the junctional complex proteins dematin and β-adducin (dematin/β-adducin double knockout mice) exhibit anisocytosis, poikilocytosis, polychromasia, and abnormal and fragmented erythrocytes with decreased lifespan [2]. Dematin/β-adducin double knockout erythrocytes also showed some membrane skeletal protein changes in common with *Hem1^−/−^* erythrocytes, including decreased spectrin, actin, and protein 4.1. Mice deficient in the junctional complex protein 4.1R also exhibit hemolytic anemia characterized by abnormal erythrocyte morphology, lowered membrane stability, and reduced levels of spectrin [47]. Our results collectively suggest that disruption of *Hem1* preferentially affects components of the junctional complex (such as actin, adducin, p55, dematin, tropomodulin, and protein 4.1), whereas components of the ankyrin complex are affected to a lesser extent (Figure 7).

Kalfa and colleagues previously found that mice deficient in Rac1 and Rac2 (*Rac1^−/−^Rac2^−/−^* mice) exhibited mild microcytic anemia with significant anisocytosis, poikilocytosis, polychromasia, and increased hypochromic cells, target cells, and fragmented erythrocytes [3], [5]. Rac-deficient erythrocytes also exhibited irregular aggregation of membrane skeletal actin and large gaps in the membrane skeleton when compared to the more uniform lattice-like structure in WT erythrocytes [3]. Rac-null erythrocyte morphology is strikingly similar to *Hem1^−/−^* erythrocytes, which is consistent with the observation that Rac1 and Rac2 act upstream of Hem-1 and the WAVE complex in other systems and cell types (see [18] for review). *Rac1^−/−^Rac2^−/−^* erythrocytes also contained increased phospho-adducin and decreased total adducin, similar to *Hem1^−/−^* erythrocytes. These collective results suggest a working model whereby Rac acts through Hem-1 to regulate F-actin polymerization and/or stability in erythrocytes. Both Rac and Hem-1 appear to inhibit phosphorylation of α-adducin at Ser724 and β-adducin at Ser713, perhaps by recruiting or stabilizing the PP2A enzyme subunits, thus strengthening the interactions between F-actin and spectrin and preventing degradation of phospho-adducin [48]. This model explains the observed increase in erythrocyte fragility and subsequent abnormal erythrocyte morphology, decreased lifespan, and anemia in both *Rac1^−/−^Rac2^−/−^* and *Hem1^−/−^* erythrocytes.

Our proteomic studies also revealed significant alterations in the representation of important metabolic enzymes within the cytosolic fractions of *Hem1^−/−^* versus WT erythrocytes. Mass spectrometry indicated statistically significant differences in the expression of 74 proteins in *Hem1^−/−^* versus wildtype erythrocytes, including enzymes involved in key biochemical events such as glycolysis. For example, Hem-1 null erythrocytes contain significantly reduced levels of phosphoglycerate kinase 1 (PGK-1), a glycolytic enzyme which is found deficient in a subset of patients afflicted with non-spherocytic haemolytic anemia [49]. In addition, the glycolytic enzyme glyceraldehyde-3-phosphate dehydrogenase (GAPDH), which is known to bind band 3 and localize to the cytoplasmic surface of the erythrocyte membrane, is also reduced in the cytosolic fraction of Hem-1 null erythrocytes relative to WT erythrocytes [50]. We speculate that disruption of the actin membrane skeleton and reduction in membrane proteins such as band 3 in Hem-1 null erythrocytes results in the release and degradation of key metabolic enzymes, leading to altered energy homeostasis. Additional investigation of the interrelationships between F-actin, membrane skeletal proteins, and key metabolic enzymes in WT and *Hem1^−/−^* erythrocytes is needed to better understand the dynamics of the erythrocyte membrane skeleton in the context of normal and abnormal actin protofilaments.

## Supporting Information

Table S1 **Proteins identified in wildtype erythrocyte insoluble fraction on LTQ-FT.**
(XLS)

Table S2 **Proteins identified in** ***Hem1^−/−^*** **erythrocyte insoluble fraction on LTQ-FT.**
(XLS)

Table S3 **Proteins detected in wildtype erythrocyte soluble fraction on LTQ-FT.**
(XLS)

Table S4 **Proteins identified in** ***Hem1^−/−^*** **erythrocyte soluble fraction on LTQ-FT.**
(XLS)

Table S5 **Peptide differences in WT and** ***Hem1^−/−^*** **erythrocyte insoluble fraction detected by CrawDad.**
(XLS)

Table S6 **Peptide differences in WT and** ***Hem1^−/−^*** **erythrocyte soluble fraction detected by CrawDad.**
(XLS)

## References

[1] Mohandas N, Gallagher PG (2008) Red cell membrane: past, present, and future. Blood 112: 3939–3948. 18988878 10.1182/blood-2008-07-161166 PMC2582001

[2] Chen H, Khan AA, Liu F, Gilligan DM, Peters LL, et al (2007) Combined deletion of mouse dematin-headpiece and beta-adducin exerts a novel effect on the spectrin-actin junctions leading to erythrocyte fragility and hemolytic anemia. J Biol Chem 282: 4124–4135. 17142833 10.1074/jbc.M610231200

[3] Kalfa TA, Pushkaran S, Mohandas N, Hartwig JH, Fowler VM, et al (2006) Rac GTPases regulate the morphology and deformability of the erythrocyte cytoskeleton. Blood 108: 3637–3645. 16882712 10.1182/blood-2006-03-005942 PMC1895472

[4] Kuhlman PA, Fowler VM (1997) Purification and characterization of an alpha 1 beta 2 isoform of CapZ from human erythrocytes: cytosolic location and inability to bind to Mg2+ ghosts suggest that erythrocyte actin filaments are capped by adducin. Biochemistry 36: 13461–13472. 9354614 10.1021/bi970601b

[5] Konstantinidis DG, George A, Kalfa TA (2010) Rac GTPases in erythroid biology. Transfus Clin Biol 17: 126–130. 20655266 10.1016/j.tracli.2010.05.002 PMC4473774

[6] Fowler VM (1996) Regulation of actin filament length in erythrocytes and striated muscle. Curr Opin Cell Biol 8: 86–96. 8791408 10.1016/s0955-0674(96)80052-4

[7] Iolascon A, Perrotta S, Stewart GW (2003) Red blood cell membrane defects. Rev Clin Exp Hematol 7: 22–56. 14692233

[8] Porro F, Costessi L, Marro ML, Baralle FE, Muro AF (2004) The erythrocyte skeletons of beta-adducin deficient mice have altered levels of tropomyosin, tropomodulin and EcapZ. FEBS Lett 576: 36–40. 15474006 10.1016/j.febslet.2004.08.057

[9] An X, Salomao M, Guo X, Gratzer W, Mohandas N (2007) Tropomyosin modulates erythrocyte membrane stability. Blood 109: 1284–1288. 17008534 10.1182/blood-2006-07-036954 PMC1785134

[10] Moyer JD, Nowak RB, Kim NE, Larkin SK, Peters LL, et al (2010) Tropomodulin 1-null mice have a mild spherocytic elliptocytosis with appearance of tropomodulin 3 in red blood cells and disruption of the membrane skeleton. Blood 116: 2590–2599. 20585041 10.1182/blood-2010-02-268458 PMC2953891

[11] Tchernia G, Mohandas N, Shohet SB (1981) Deficiency of skeletal membrane protein band 4.1 in homozygous hereditary elliptocytosis. Implications for erythrocyte membrane stability. J Clin Invest 68: 454–460. 6894932 10.1172/JCI110275 PMC370818

[12] Burkhardt JK, Carrizosa E, Shaffer MH (2008) The actin cytoskeleton in T cell activation. Annu Rev Immunol 26: 233–259. 18304005 10.1146/annurev.immunol.26.021607.090347

[13] Mulloy JC, Cancelas JA, Filippi MD, Kalfa TA, Guo F, et al (2010) Rho GTPases in hematopoiesis and hemopathies. Blood 115: 936–947. 19965643 10.1182/blood-2009-09-198127 PMC2817638

[14] Boukharov AA, Cohen CM (1998) Guanine nucleotide-dependent translocation of RhoA from cytosol to high affinity membrane binding sites in human erythrocytes. Biochem J 330 (Pt 3): 1391–1398. 10.1042/bj3301391 PMC1219287 9494111

[15] Jaffe AB, Hall A (2005) Rho GTPases: biochemistry and biology. Annu Rev Cell Dev Biol 21: 247–269. 16212495 10.1146/annurev.cellbio.21.020604.150721

[16] Park H, Staehling-Hampton K, Appleby MW, Brunkow ME, Habib T, et al (2008) A point mutation in the murine Hem1 gene reveals an essential role for Hematopoietic protein 1 in lymphopoiesis and innate immunity. J Exp Med 205: 2899–2913. 19015308 10.1084/jem.20080340 PMC2585840

[17] Takenawa T, Suetsugu S (2007) The WASP-WAVE protein network: connecting the membrane to the cytoskeleton. Nat Rev Mol Cell Biol 8: 37–48. 17183359 10.1038/nrm2069

[18] Park H, Chan MM, Iritani BM (2010) Hem-1: putting the “WAVE” into actin polymerization during an immune response. FEBS Lett 584: 4923–4932. 20969869 10.1016/j.febslet.2010.10.018 PMC3363972

[19] Pollitt AY, Insall RH (2009) WASP and SCAR/WAVE proteins: the drivers of actin assembly. J Cell Sci 122: 2575–2578. 19625501 10.1242/jcs.023879 PMC2954249

[20] Baumgartner S, Martin D, Chiquet-Ehrismann R, Sutton J, Desai A, et al (1995) The HEM proteins: a novel family of tissue-specific transmembrane proteins expressed from invertebrates through mammals with an essential function in oogenesis. J Mol Biol 251: 41–49. 7643388 10.1006/jmbi.1995.0414

[21] Beutler E, West C, Blume KG (1976) The removal of leukocytes and platelets from whole blood. J Lab Clin Med 88: 328–333. 956688

[22] Iritani BM, Forbush KA, Farrar MA, Perlmutter RM (1997) Control of B cell development by Ras-mediated activation of Raf. EMBO J 16: 7019–7031. 9384581 10.1093/emboj/16.23.7019 PMC1170305

[23] Pfaffl MW (2001) A new mathematical model for relative quantification in real-time RT-PCR. Nucleic Acids Res 29: e45. 11328886 10.1093/nar/29.9.e45 PMC55695

[24] Finney GL, Blackler AR, Hoopmann MR, Canterbury JD, Wu CC, et al (2008) Label-free comparative analysis of proteomics mixtures using chromatographic alignment of high-resolution muLC-MS data. Anal Chem 80: 961–971. 18189369 10.1021/ac701649e

[25] Wooden JM, Finney GL, Rynes E, Maccoss MJ, Lambert AJ, et al (2011) Comparative proteomics reveals deficiency of SLC9A1 (sodium/hydrogen exchanger NHE1) in beta-adducin null red cells. Br J Haematol 154: 492–501. 21689084 10.1111/j.1365-2141.2011.08612.x PMC4515348

[26] Ringrose JH, van Solinge WW, Mohammed S, O’Flaherty MC, van Wijk R, et al (2008) Highly efficient depletion strategy for the two most abundant erythrocyte soluble proteins improves proteome coverage dramatically. J Proteome Res 7: 3060–3063. 18494517 10.1021/pr8001029

[27] Chen K, Liu J, Heck S, Chasis JA, An X, et al (2009) Resolving the distinct stages in erythroid differentiation based on dynamic changes in membrane protein expression during erythropoiesis. Proc Natl Acad Sci U S A 106: 17413–17418. 19805084 10.1073/pnas.0909296106 PMC2762680

[28] Sents W, Ivanova E, Lambrecht C, Haesen D, Janssens V (2012) The biogenesis of active protein phosphatase 2A holoenzymes: a tightly regulated process creating phosphatase specificity. FEBS J. 10.1111/j.1742-4658.2012.08579.x 22443683

[29] Shi Y (2009) Serine/threonine phosphatases: mechanism through structure. Cell 139: 468–484. 19879837 10.1016/j.cell.2009.10.006

[30] Matsuoka Y, Li X, Bennett V (1998) Adducin is an in vivo substrate for protein kinase C: phosphorylation in the MARCKS-related domain inhibits activity in promoting spectrin-actin complexes and occurs in many cells, including dendritic spines of neurons. J Cell Biol 142: 485–497. 9679146 10.1083/jcb.142.2.485 PMC2133059

[31] Barkalow KL, Italiano JE Jr, Chou DE, Matsuoka Y, Bennett V, et al (2003) Alpha-adducin dissociates from F-actin and spectrin during platelet activation. J Cell Biol 161: 557–570. 12743105 10.1083/jcb.200211122 PMC2172952

[32] Garcia S, DiSanto J, Stockinger B (1999) Following the development of a CD4 T cell response in vivo: from activation to memory formation. Immunity 11: 163–171. 10485651 10.1016/s1074-7613(00)80091-6

[33] Weiner OD, Rentel MC, Ott A, Brown GE, Jedrychowski M, et al (2006) Hem-1 complexes are essential for Rac activation, actin polymerization, and myosin regulation during neutrophil chemotaxis. PLoS Biol 4: e38. 16417406 10.1371/journal.pbio.0040038 PMC1334198

[34] Bompard G, Caron E (2004) Regulation of WASP/WAVE proteins: making a long story short. J Cell Biol 166: 957–962. 15452139 10.1083/jcb.200403127 PMC2172026

[35] Harwood NE, Batista FD (2011) The cytoskeleton coordinates the early events of B-cell activation. Cold Spring Harb Perspect Biol 3. 10.1101/cshperspect.a002360 PMC3039531 21047917

[36] Bokoch GM (2005) Regulation of innate immunity by Rho GTPases. Trends Cell Biol 15: 163–171. 15752980 10.1016/j.tcb.2005.01.002

[37] Bruce LJ, Beckmann R, Ribeiro ML, Peters LL, Chasis JA, et al (2003) A band 3-based macrocomplex of integral and peripheral proteins in the RBC membrane. Blood 101: 4180–4188. 12531814 10.1182/blood-2002-09-2824

[38] Gilligan DM, Sarid R, Weese J (2002) Adducin in platelets: activation-induced phosphorylation by PKC and proteolysis by calpain. Blood 99: 2418–2426. 11895774 10.1182/blood.v99.7.2418

[39] van de Water B, Tijdens IB, Verbrugge A, Huigsloot M, Dihal AA, et al (2000) Cleavage of the actin-capping protein alpha -adducin at Asp-Asp-Ser-Asp633-Ala by caspase-3 is preceded by its phosphorylation on serine 726 in cisplatin-induced apoptosis of renal epithelial cells. J Biol Chem 275: 25805–25813. 10823823 10.1074/jbc.M001680200

[40] Baharians Z, Schonthal AH (1998) Autoregulation of protein phosphatase type 2A expression. J Biol Chem 273: 19019–19024. 9668082 10.1074/jbc.273.30.19019

[41] Ruediger R, Brewis N, Ohst K, Walter G (1997) Increasing the ratio of PP2A core enzyme to holoenzyme inhibits Tat-stimulated HIV-1 transcription and virus production. Virology 238: 432–443. 9400615 10.1006/viro.1997.8873

[42] Slater SJ, Seiz JL, Stagliano BA, Stubbs CD (2001) Interaction of protein kinase C isozymes with Rho GTPases. Biochemistry 40: 4437–4445. 11284700 10.1021/bi001654n

[43] Suzuki K, Chikamatsu Y, Takahashi K (2005) Requirement of protein phosphatase 2A for recruitment of IQGAP1 to Rac-bound beta1 integrin. J Cell Physiol 203: 487–492. 15521075 10.1002/jcp.20249

[44] Khan AA, Hanada T, Mohseni M, Jeong JJ, Zeng L, et al (2008) Dematin and adducin provide a novel link between the spectrin cytoskeleton and human erythrocyte membrane by directly interacting with glucose transporter-1. J Biol Chem 283: 14600–14609. 18347014 10.1074/jbc.M707818200 PMC2386908

[45] Salomao M, Zhang X, Yang Y, Lee S, Hartwig JH, et al (2008) Protein 4.1R-dependent multiprotein complex: new insights into the structural organization of the red blood cell membrane. Proc Natl Acad Sci U S A 105: 8026–8031. 18524950 10.1073/pnas.0803225105 PMC2430353

[46] Lach A, Grzybek M, Heger E, Korycka J, Wolny M, et al (2012) Palmitoylation of MPP1 (membrane-palmitoylated protein 1)/p55 is crucial for lateral membrane organization in erythroid cells. J Biol Chem 287: 18974–18984. 22496366 10.1074/jbc.M111.332981 PMC3365931

[47] Shi ZT, Afzal V, Coller B, Patel D, Chasis JA, et al (1999) Protein 4.1R-deficient mice are viable but have erythroid membrane skeleton abnormalities. J Clin Invest 103: 331–340. 9927493 10.1172/JCI3858 PMC407893

[48] George A, Pushkaran S, Li L, An X, Zheng Y, et al (2010) Altered phosphorylation of cytoskeleton proteins in sickle red blood cells: the role of protein kinase C, Rac GTPases, and reactive oxygen species. Blood Cells Mol Dis 45: 41–45. 20231105 10.1016/j.bcmd.2010.02.006 PMC2878931

[49] Beutler E (2007) PGK deficiency. Br J Haematol 136: 3–11. 17222195 10.1111/j.1365-2141.2006.06351.x

[50] Campanella ME, Chu H, Wandersee NJ, Peters LL, Mohandas N, et al (2008) Characterization of glycolytic enzyme interactions with murine erythrocyte membranes in wild-type and membrane protein knockout mice. Blood 112: 3900–3906. 18698006 10.1182/blood-2008-03-146159 PMC2572807

